# Predictors for successful weight reduction during treatment with Dapagliflozin among patients with type 2 diabetes mellitus in primary care

**DOI:** 10.1186/s12875-022-01748-1

**Published:** 2022-05-27

**Authors:** Youn Huh, Young Sik Kim

**Affiliations:** 1 Department of Family Medicine, Uijeongbu Eulji Medical Center, Eulji Unversity, Gyeonggi-do, Republic of Korea; 2 Department of Family Medicine, Asan Medical Center, University of Ulsan College of Medicine, 88 Olymphic-Ro 43-Gil, Songpa-gu, Seoul, 05505 Republic of Korea

**Keywords:** Diabetes mellitus, Sodium-glucose transporter 2 inhibitor, Weight loss, Primary health care

## Abstract

**Aims:**

Studies on factors affecting weight loss effect after start of dapagliflozin in type 2 diabetes mellitus (T2DM) patients are few. The aim of this study was to identify if there were any patient characteristics that could predict weight loss after starting treatment with dapagliflozin.

**Methods:**

The study included 200 Korean patients with T2DM who were prescribed dapagliflozin in a family medicine clinic during 2014–2019. We studied patients for 1 year after starting dapagliflozin treatment. Data were collected from medical records. Clinically meaningful weight reduction was defined as ≥3% decrease in body weight and odds ratios (ORs) and 95% confidence intervals (CIs) for succeeding this weight reduction was calculated for different baseline characteristics.

**Results:**

In total, 113 (56.5%) patients were male. Weight loss of ≥3% in 1 year treatment with dapagliflozin was achieved in 122 (61%) patients. The likelihood of this level of weight loss was significantly increased with regular exercise (OR 2.13, 95% CI 1.07–4.25), with concomitant metformin treatment (OR 2.90, 95% CI 1.23–6.80), and in patients with normal renal function (OR 13.84, 95% CI 1.33–144.26). Patients receiving sulfonylurea treatment were less likely to achieve ≥3% weight reduction (OR 0.39, 95 CI 0.19–0.79).

**Conclusions:**

T2DM patients that performed regular exercise, had normal renal function and were receiving metformin were more likely to have clinically meaningful body weight reduction after one year treatment with dapagliflozin.

## Background

Type 2 diabetes mellitus (T2DM) is one of the most common chronic diseases. Its prevalence is expected to increase to up to 439 million by 2030 [1]. It has been reported that ≥80% of T2DM patients live in developing countries, with ≥60% living in Asia; 13.7% (4.8 million) of South Korean adults aged ≥30 years had T2DM in 2014 [2, 3]. T2DM is strongly associated with an increased risk of cardiovascular diseases (CVDs), renal failure, blindness, and mortality [4]. Thus, the worldwide socioeconomic burden of T2DM is substantial. Age ≥ 40 years, and a family history of diabetes, vascular disease, hypertension, or dyslipidemia are recognized risk factors of T2DM.

Weight loss by lifestyle changes is the first-line therapy for T2DM because of the expected improvement in glycemic control and other associated risk factors [5]. T2DM patients who have lost weight are more likely to achieve the target glycated hemoglobin (HbA1c) values than those who maintained or gained weight [6]. The results of a meta-analysis on the effect of weight loss on lifestyle modifications in obese T2DM patients revealed that a weight loss of > 5% significantly improved their fasting blood glucose, lipid levels, and blood pressure (BP) [7]. A weight loss of 3% is effective in improving blood pressure, fasting blood glucose, and cholesterol [8]. The microvascular complications of T2DM include retinopathy, nephropathy, and peripheral and autonomic neuropathy. The risk of developing these complications is associated not only with the duration of DM, blood glucose control, and BP, but also with obesity [9, 10].

There are many pharmacotherapeutic options available for controlling glycemia in patients with T2DM. Sodium glucose co-transporter 2 (SGLT2) inhibitors are oral glucose-lowering drugs associated with weight reduction [11]. Therefore, they are a useful option for overweight T2DM patients. Dapagliflozin was one of the first SGLT2 inhibitors introduced in Korea.

However, studies on the effects of dapagliflozin on weight loss in T2DM patients in primary care settings are still few. In addition, there is a lack of studies on primary care patients who may benefit more from dapagliflozin treatment in terms of its weight loss effects. Thus, this study aimed to investigate to find predictors for ≥3% weight loss during treatment with dapagliflozin among Korean patients with T2DM seeking primary care treatment in Korea.

## Methods

### Data source and study population

A total of 433 Korean adults who were prescribed dapagliflozin at the Department of Family Medicine in Asan Medical Center between January 1, 2014, and April 30, 2019, were enrolled in the study. The exclusion criteria were as follows: patients with missing data (*n* = 72), those admitted to other departments such as internal medicine (*n* = 65), and those whose duration of treatment with dapagliflozin was < 1 year (*n* = 96). Finally, 200 participants (113 men and 87 women) were included in the study.

This study adhered to the principles of the Declaration of Helsinki. The Institutional Review Board of Asan Medical Center approved our study [No. AMC-IRB 2020–0824]. The requirement for informed consent was waived by the Institutional Review Board of Asan Medical Center because of the retrospective nature of the study.

### Covariates

Participants’ general characteristics, including sex, age, concomitant medications, comorbid diseases, lifestyle, vital signs, laboratory examinations, and anthropometry, were obtained from their medical records (Asan Biomedical Research Environment) and analyzed.

The patients’ age at initiation of dapagliflozin was annotated, and height was measured to the nearest 0.1 cm using a stadiometer (SECA 225, Hamburg, Germany). Body weight was measured to the nearest 0.1 kg using a balance scale (GL-6000-20, Cas, Yangju, Korea) with the participants wearing a lightweight gown or underwear. A significant weight loss was defined as ≥3% decrease in body weight after 1 year of dapagliflozin treatment. On the other hand, when the weight reduction was < 3%, we considered it as no change in body weight. An increase in body weight of > 3% was termed as weight gain. Body mass index (BMI) was calculated by dividing the patients’ weight (kg) by the square of their height (m). Next, we divided the participants into BMI quartile groups, and the cutoffs were 26.40, 27.88, and 30.11 kg/m^2^. Lifestyle-related factors such as alcohol consumption, smoking, and exercise habits were evaluated using a self-reported questionnaire, which was constructed by the authors. This was completed by the patients on their first visit. Using this questionnaire, the patients’ alcohol consumption was assessed. They were asked whether they drank alcohol, and the frequency of alcohol consumption per week, and the number of alcoholic drinks consumed per day were evaluated. Men who consumed ≥2 drinks per day and women who consumed ≥1 drink per day were referred to as heavy alcohol drinkers [12]. Patients were classified based on their smoking history as non-smokers or current smokers. Moreover, regular exercise was also included in the questionnaire. Regular exercise was defined as exercising ≥3 times per week. Information on pertinent medical history, such as hypertension, dyslipidemia, and cardiovascular diseases, based on the medication prescribed in Asan Medical Center was collected. In addition, information regarding the use of other antihyperglycemic agents, such as metformin and sulfonylurea (SU), was collected.

Blood samples were obtained after an overnight fast, and serum levels of glucose, HbA1c, and creatinine were measured. The estimated glomerular filtration rate (eGFR) was calculated using the following formula developed in the Modification of Diet in Renal Disease study: eGFR = 175 × serum creatinine^− 1.154^ × age^− 0.203^ (multiplied by 0.742 in women) [13]. Abnormal renal function was defined as eGFR < 60 mL/min/1.73 m^2^, and normal renal function was defined as eGFR ≥60 mL/min/1.73 m^2^.

### Statistical analysis

Statistical analyses were conducted using SPSS v. 24.0 (IBM Corp., Armonk, NY, USA). Baseline characteristics of the study participants are presented as mean ± standard deviation for continuous variables and number (percentage) for categorical variables. The values were compared using analysis of variance for continuous variables and chi-squared test for categorical variables. A paired T-test was performed to confirm the change in body weight, BMI, and fasting glucose and HbA1c levels before and after the use of dapagliflozin. We conducted a multivariable logistic regression analysis and calculated the odds ratio (OR) and 95% confidence intervals (CIs), and multivariable logistic regression analysis was performed to evaluate the factors associated with a weight loss of ≥3%. Model 1 was unadjusted, and model 2 was adjusted for age, sex, alcohol consumption, smoking status, physical activity, hypertension, dyslipidemia, CVD, metformin, SU, and renal function. A *p-*value *<* 0.05 was considered statistically significant.

## Results

### Baseline characteristics

Table 1 shows the participants’ baseline characteristics. Among the 200 participants, 113 (56.5%) were men. The mean age was 60.9 years, mean BMI was 28.1 kg/m2 and the mean HbA1c and fasting blood glucose levels were 7.75% and 158.41 mg/dL respectively. Mean eGFR was 89.6 mL/min/1.73 m2. Females were slightly older or 63.3 years vs. 59.0 years for males and had higher fasting glucose level (154.1 vs 131.8 mg/dL). The proportion of current smokers, heavy drinkers, and patients who regularly exercised was 21.0, 32.5, and 33.5%, respectively; these proportions were higher in men than in women. The rates of hypertension, dyslipidemia, and CVD were 75.5, 91.5, and 3.5%, respectively, and there was no difference between men and women. The proportion of patients taking metformin and SU was 78.5 and 40.0%, respectively, and there was no difference between men and women.

**Table 1:** Demographic profile and baseline characteristics of the study participants

		Total (*n* = 200)	Male (*n* = 113)	Female (*n* = 87)
Age (years)	Mean (SD)	60.89 ± 10.08	59.03 ± 9.99	63.31 ± 9.73
Weight (kg)	Mean (SD)	73.77 ± 11.70	78.23 ± 11.39	67.97 ± 9.37
Height (cm)	Mean (SD)	162.40 ± 8.92	168.11 ± 6.40	154.98 ± 5.67
BMI (kg/m^2^)	Mean (SD)	28.05 ± 2.96	27.90 ± 2.65	28.26 ± 3.33
BMI (kg/m^2^)	Lower quartile	26.40	26.15	26.50
	Median	27.88	27.64	28.40
	Upper quartile	30.11	29.73	30.46
HbA1c (%)	Mean (SD)	7.75 ± 1.11	7.77 ± 1.13	7.74 ± 1.10
Glucose (mg/dL)	Mean (SD)	158.41 ± 45.93	131.77 ± 51.36	154.05 ± 37.58
GFR (mL/min/1.73 m^2^)	Mean (SD)	89.60 ± 15.56	89.54 ± 16.12	89.67 ± 14.89
Smoking status	Current smoker	42 (21.0%)	39 (34.5%)	3 (3.4%)
Alcohol consumption	Heavy drinker	65 (32.5%)	55 (48.7%)	10 (11.5%)
Exercise	Regular exercise	67 (33.5%)	44 (38.9%)	23 (26.4%)
Comorbidity	Hypertension	151 (75.5%)	88 (77.9%)	63 (72.4%)
	Dyslipidemia	183 (91.5%)	101 (89.4%)	82 (94.3%)
	CVD	7 (3.5%)	7 (6.2%)	0 (0%)
Co-DM medication	Metformin	157 (78.5%)	92 (81.4%)	65 (74.7%)
	Sulfonylurea	80 (40.0%)	43 (38.1%)	37 (42.5%)

Data are presented as mean ± SD or number (percent)

*Abbreviations*: *SD* Standard deviation, *BMI* Body mass index, *HbA1c* Glycated hemoglobin, *GFR* Glomerular filtration rate, *CVD* Cardiovascular disease, *DM* Diabetes mellitus

### Mean change from baseline in body weight, BMI, and fasting blood glucose and HbA1c levels

Table 2 shows the mean change from baseline in body weight, BMI, fasting blood glucose and HbA1c levels. After 1 year of treatment with dapagliflozin, participants’ mean body weight decreased from 74.76 kg to 71.37 kg (*p* < 0.001), and BMI decreased from 27.95 to 27.02 kg/m2 (*p* < 0.001). Additionally, the mean fasting blood glucose and HbA1c levels decreased from 150.88 mg/dL and 7.33% to 136.93 mg/dL and 7.22%, respectively (*p* < 0.001). The reductions in body weight, BMI, and fasting blood glucose were statistically significant following the use of dapagliflozin in the total population as well as in the male and female subgroups. The reductions in HbA1c were not statistically significant in the male and female subgroups.

**Table 2:** Mean changes from baseline in body weight, BMI, fasting blood glucose, and HbA1c levels

		Total (*n* = 200)			Male (*n* = 113)			Female (n = 87)		
		M	SD	*p*-value	M	SD	*p*-value	M	SD	*p*-value
Body weight (kg)	Start of treatment	74.76	16.72	< 0.001	80.34	19.01	0.005	67.53	9.08	< 0.001
	After 1 year of treatment	71.37	10.59		75.98	9.17		65.39	9.28	
BMI (kg/m^2^)	Start of treatment	27.95	2.96	< 0.001	27.85	2.72	< 0.001	28.09	3.25	< 0.001
	After 1 year of treatment	27.02	3.07		26.90	2.81		27.18	3.40	
Glucose (mg/dL)	Start of treatment	150.88	29.74	< 0.001	151.56	28.26	< 0.001	150.00	31.71	< 0.001
	After 1 year of treatment	136.93	24.10		139.10	24.79		134.11	23.00	
HbA1c (%)	Start of treatment	7.33	0.83	0.037	7.29	0.75	0.234	7.38	0.92	0.076
	After 1 year of treatment	7.22	0.77		7.21	0.79		7.24	0.75	

*Abbreviations*: *HbA1c* Glycated hemoglobin, *BMI* Body mass index, *SD* Standard deviation

### Factors associated with weight loss after dapagliflozin use

Table 3 describes the population that achieved a weight loss of ≥3% after 1 year of dapagliflozin treatment. Sixty-one percent of the participants had weight reduction of ≥3%. This level of weight reduction was significantly more likely to happen in patients who exercised regularly or had normal kidney function.

**Table 3:** Factors associated with weight loss after dapagliflozin use

		weight loss of ≥3% (*N* = 122)	*p*-value
Sex	Male	71 (62.8%)	0.55
	Female	51 (58.6%)	
BMI	Q1	29 (58.0%)	0.71
	Q2	28 (56.0%)	
	Q3	32 (65.3%)	
	Q4	33 (64.7%)	
Alcohol consumption	Heavy drinker^a^ (−)	79 (58.5%)	0.30
	Heavy drinker^a^ (+)	43 (66.2%)	
Smoking status	Non-smoker	98 (62.0%)	0.56
	Current smoker	24 (57.1%)	
Regular exercise^b^	No	74 (55.6%)	0.03
	Yes	48 (71.6%)	
Hypertension	No	30 (61.2%)	0.97
	Yes	92 (60.9%)	
Dyslipidemia	No	12 (70.6%)	0.40
	Yes	110 (60.1%)	
CVD	No	117 (60.6%)	0.57
	Yes	5 (71.4%)	
Metformin	No	23 (53.5%)	0.25
	Yes	99 (63.1%)	
Sulfonylurea	No	79 (65.8%)	0.09
	Yes	43 (53.8%)	
Renal function	eGFR < 60 mL/min/1.73 m^2^	1 (16.7%)	0.02
	eGFR ≥60 mL/min/1.73 m^2^	121 (62.4%)	

Data are presented as number (percent)

^a^Heavy drinker was defined as men who consumed ≥2 drinks per day or women who consumed ≥1 drink per day

^b^Regular exercise was defined as exercising three or more times per week

*Abbreviations*: *BMI* Body mass index, *HbA1c* Glycated hemoglobin, *GFR* Glomerular filtration rate, *CVD* Cardiovascular disease, *DM* Diabetes mellitus

Table 4 shows the factors analyzed for association with weight loss of ≥3% after 1 year of dapagliflozin administration. The likelihood achieving this level of weight loss was significantly higher with regular exercises (OR: 2.16, 95% CI: 1.07–4.36 in model 2), in patients receiving metformin treatment (OR: 2.90, 95% CI: 1.23–6.80 in model 2), and in patients with normal renal (OR: 13.84, 95% CI: 1.33–144.26 in model 2). On the other hand, this was significantly less likely to happen in patients who took SU (OR: 0.39, 95% CI: 0.19–0.79 in model 2). Age, sex, BMI, alcohol consumption status, smoking status, hypertension, dyslipidemia, and CVD were not associated with weight loss after dapagliflozin use.

**Table 4:** A logistic regression analysis for predictors of ≥3% weight loss

		Model 1	Model 2
		OR (95% CI)	OR (95% CI)
Age	Per 1 year	0.99 (0.96–1.01)	1.01 (0.97–1.04)
Sex	Male	1(ref.)	1(ref.)
	Female	0.84 (0.47–1.49)	1.08 (0.52–2.24)
BMI	Q1	1(ref.)	1(ref.)
	Q2	0.92 (0.42–2.04)	0.72 (0.29–1.76)
	Q3	1.36 (0.60–3.07)	0.73 (0.30–1.76)
	Q4	1.33 (0.60–2.96)	1.14 (0.47–2.81)
Alcohol consumption	Heavy drinker^a^ (−)	1(ref.)	1(ref.)
	Heavy drinker^a^ (+)	1.39 (0.75–2.57)	1.35 (0.65–2.80)
Smoking status	Non-smoker	1 (ref)	1(ref.)
	Current smoker	1.23 (0.61–2.44)	1.09 (0.46–2.55)
Regular exercise^b^	No	1 (ref)	1(ref.)
	Yes	2.01 (1.07–3.79)	2.16 (1.07–4.36)
Hypertension	No	1 (ref)	1(ref.)
	Yes	0.99 (0.51–1.91)	0.90 (0.42–1.90)
Dyslipidemia	No	1 (ref)	1(ref.)
	Yes	0.63 (0.21–1.86)	0.65 (0.20–2.09)
CVD	No	1 (ref)	1(ref.)
	Yes	1.62 (0.31–8.58)	1.69 (0.23–12.30)
Metformin	No	1 (ref)	1(ref.)
	Yes	1.48 (0.75–2.93)	2.90 (1.23–6.80)
Sulfonylurea	No	1 (ref)	1(ref.)
	Yes	0.60 (0.34–1.08)	0.39 (0.19–0.79)
Renal function	eGFR < 60 mL/min/1.73 m^2^	1 (ref)	1(ref.)
	eGFR ≥60 mL/min/1.73 m^2^	8.29 (0.95–72.34)	13.84 (1.33–144.26)

Values were calculated by multivariable linear regression analyses

Model 1 was unadjusted

Mod1 2 was adjusted for age, sex, BMI, alcohol consumption status, smoking status, physical activity, hypertension, dyslipidemia, CVD, metformin, sulfonylurea, and renal function

^a^Heavy drinker was defined as men who consumed ≥2 drinks per day or women who consumed ≥1 drink per day

^b^Regular exercise was defined as exercising three or more times per week

*Abbreviations*: *OR* Odds ratio, *CI* Confidence interval, *BMI* Body mass index, *CVD* Cardiovascular disease, *GFR* Glomerular filtration rate

## Discussion

Our study showed that when starting dapagliflozin treatment, patients that exercised regularly, had normal kidney function or were treated with metformin were more likely to have a clinically meaningful weight reduction after start of dapagliflozin treatment. On the other hand, patients treated with sulfonylurea were less likely to do so. These results could help physicians choose the appropriate DM medication for these patients.

Obesity is an independent risk factor for hypertension and dyslipidemia, as well as CVD, which is the major cause of death in patients with T2DM. Weight loss is considered key in T2DM management because it leads to a potential reduction in blood glucose levels. In addition, weight loss can have an impact on other health problems commonly associated with T2DM, thereby reducing the need for medications, not only for hyperglycemia but also for hypertension and hyperlipidemia [14]. In a prospective analysis of 4970 overweight individuals with DM who underwent a 12-year follow-up, those with weight loss had a 25% reduction in total mortality and a 28% reduction in CVD- and diabetes mellitus-related mortality compared with individuals who reported no changes in weight [15]. Although the role of weight loss in reducing the potential risk of complications in T2DM is unclear, there is evidence of the beneficial effects of weight loss in reducing proteinuria in overweight and obese patients. In patients with non-diabetic renal disease with nephropathy, a weight loss of approximately 4% of the initial body weight is associated with a 31.2% ± 37% decrease of proteinuria from the baseline value [16]. Weight loss could improve the quality of life of T2DM patients and common comorbidities, such as obstructive sleep apnea and depression [17–19].

Many T2DM patients have problems of weight gain, secondary to intake of drugs such as SUs, insulin, glinides, and thiazolidinediones [20, 21]. Most of the other classes of antihyperglycemic agents are body-weight neutral, besides orally administered SGLT2 inhibitors, and subcutaneously administered GLP-1 receptor agonists and amylin agonists, which are associated with weight loss [21]. The administration of an SGLT2 inhibitor improves glycemic control and reduces body weight, fat mass, and adipose tissue volume [22–24]. Dapagliflozin, an SGLT2 inhibitor widely used in clinical practice, exerts its glucose-lowering effects by inhibiting the SGLT2 protein in the proximal tubule of the kidney, resulting in the excretion of glucose and extra calories into the urine [25]. In individuals with T2DM who received dapagliflozin doses between 2.5 and 20 mg, the 24-hr glucose excretion after 1 and 14 days ranged from 38 g to 77 g and from 42 g to 73 g, respectively [26]. The use of SGLT2 inhibitors alone has been known to reduce HbA1c by 0.5–1%. However, the participants in our study had decreased HbA1c by 0.11%, which is thought to be less effective in reducing HbA1c by using SGLT2 inhibitors in overweight or obese patients with poor control of T2DM. Some studies showed that dapagliflozin is effective in reducing HbA1c, body weight, and blood pressure in a real-world primary care setting in UK [27] and Germany [28]. T2DM patients frequently exhibit increased ectopic fat accumulation [29] and a progressive decline in muscle mass and quality [30], and it is by fat mass reduction that treatment with SGLT2 inhibitors reduces body weight [22]. One study showed that the weight loss caused by dapagliflozin in T2DM patients is primarily the result of a reduction in fat mass than lean mass [31]. SGLT2 inhibitors induce gluconeogenesis, lipolysis, and increase glucagon levels [32, 33], which may also contribute to fat mass reduction. This might positively affect the patients’ quality of life, treatment satisfaction, and motivation for the continuation of anti-diabetic therapy [34].

Regular exercise, renal function, and metformin use were associated with weight loss after dapagliflozin treatment. Exercise contributes to weight loss, improves blood glucose control in T2DM, reduces cardiovascular risk factors, and improves general well-being [35]. The mechanism of action of dapagliflozin is dependent on renal function. Clinical trials with dapagliflozin have been primarily conducted in patients with normal renal function or those with mild renal impairment [36], because the efficacy of dapagliflozin is reduced in patients with significant renal impairment. In addition, urinary glucose excretion decreased with increasing renal impairment and correlated with decreased GFR and renal clearance [37]. In our study, we found that the effect of dapagliflozin on weight loss was better in patients with normal renal function than in those with decreased renal function. In our study, there were only a few patients with decreased renal function due to the small number of participants, and the interpretation of the results was limited accordingly. In the future, large-scale research is needed. When metformin was prescribed alone, there was no weight loss effect. However, when dapagliflozin was prescribed in T2DM patients taking metformin, the weight reduction effect was better than that of patients not taking metformin. According to the current guideline for T2DM in Korea [38], metformin is the first drug recommended for T2DM; thus, we found that patients taking metformin will benefit more with additional dapagliflozin treatment. Given that SU closes the KATP channels on β-cell plasma membranes and increases insulin secretion, it induces weight gain in patients with T2DM [20, 21]. Considering this mechanism, the weight loss effect of dapagliflozin may be reduced when administered in combination with an SU.

Our study had several limitations. First, although the study sample size was determined through studies conducted similar to our study [39, 40], the number of participants was small. Secondly, for the assessment of comorbidities, we solely relied on the information in the patient’s medical records and medications prescribed at our hospital. In our study, the weight loss effect of dapagliflozin was observed after 1 year, but no long-term effects were evaluated. Weight loss plateaued at approximately 6–12 months; hence, 12 months of dapagliflozin treatment may be sufficient for maximal weight loss [41]. There was no valid information regarding the onset of DM. Given that T2DM and hypertension of our study participants were managed in a primary care setting, there were limitations in comparing the fasting blood glucose and HbA1c levels and BP before and after dapagliflozin treatment. Although we might have considered some of the factors that influenced weight loss in T2DM patients, we could not account for all confounding variables. Finally, our study was not a double-blinded, placebo-controlled trial. Therefore, we were unable to compare the effects of dapagliflozin with those of other hyperglycemic agents. Despite these limitations, our study involved T2DM patients in a Korean primary care setting. In addition to describing the weight loss with dapagliflozin in primary care conditions, we also identified patients who are more likely to have clinically meaningful weight reduction after start of dapagliflozin treatment.

## Conclusions

We showed that dapagliflozin can effectively reduce weight in T2DM patients who are regularly exercising, having a normal kidney function, and combining metformin. However, T2DM patients taking SU were less likely to have meaningful weight reduction. In T2DM patients with these conditions, it is possible to consider the preferential use of SGLT2 inhibitors over other antihyperglycemic agents.

## Data Availability

The datasets generated during and/or analyzed during the current study are available from the corresponding author on reasonable request.

## Abbreviations

T2DM: T2 diabetes mellitus
OR: Odds ratios
CT: Confidence intervals
CVD: Cardiovascular diseases
HbA1c: Glycated hemoglobin
SGLT2: Sodium glucose co-transporter 2
SU: Sulfonylurea
eGFR: Estimated glomerular filtration rate
